# Genomic Characterization of Multidrug-Resistant Enterobacteriaceae Clinical Isolates from Southern Thailand Hospitals: Unraveling Antimicrobial Resistance and Virulence Mechanisms

**DOI:** 10.3390/antibiotics13060531

**Published:** 2024-06-06

**Authors:** Thunchanok Yaikhan, Sirikan Suwannasin, Kamonnut Singkhamanan, Sarunyou Chusri, Rattanaruji Pomwised, Monwadee Wonglapsuwan, Komwit Surachat

**Affiliations:** 1 Department of Biomedical Sciences and Biomedical Engineering, Faculty of Medicine, Prince of Songkla University, Songkhla 90110, Thailand; ythuncha@medicine.psu.ac.th (T.Y.); sirikan4036@gmail.com (S.S.); skamonnu@medicine.psu.ac.th (K.S.); 2 Division of Infectious Diseases, Department of Internal Medicine, Faculty of Medicine, Prince of Songkla University, Songkhla 90110, Thailand; 3 Division of Biological Science, Faculty of Science, Prince of Songkla University, Songkhla 90110, Thailand; rattanaruji.p@psu.ac.th (R.P.); monwadee.wo@psu.ac.th (M.W.); 4 Translational Medicine Research Center, Faculty of Medicine, Prince of Songkla University, Songkhla 90110, Thailand

**Keywords:** Enterobacteriaceae, antimicrobial resistance gene, virulence factor, plasmid

## Abstract

The emergence and spread of antimicrobial resistance (AMR) among Enterobacteriaceae pose significant threats to global public health. In this study, we conducted a short-term surveillance effort in Southern Thailand hospitals to characterize the genomic diversity, AMR profiles, and virulence factors of Enterobacteriaceae strains. We identified 241 carbapenem-resistant Enterobacteriaceae, of which 12 were selected for whole-genome sequencing (WGS) and genome analysis. The strains included *Proteus mirabilis*, *Serratia nevei*, *Klebsiella variicola*, *Klebsiella aerogenes*, *Klebsiella indica*, *Klebsiella grimontii*, *Phytobacter ursingii*, *Phytobacter palmae*, *Kosakonia* spp., and *Citrobacter freundii*. The strains exhibited high levels of multidrug resistance, including resistance to carbapenem antibiotics. Whole-genome sequencing revealed a diverse array of antimicrobial resistance genes (ARGs), with strains carrying genes for ß-lactamase, efflux pumps, and resistance to other antibiotic classes. Additionally, stress response, metal tolerance, and virulence-associated genes were identified, highlighting the adaptability and pathogenic potential of these strains. A plasmid analysis identified several plasmid replicons, including IncA/C2, IncFIB(K), and Col440I, as well as several plasmids identical to those found globally, indicating the potential for the horizontal gene transfer of ARGs. Importantly, this study also identified a novel species of *Kosakonia* spp. PSU27, adding to the understanding of the genetic diversity and resistance mechanisms of Enterobacteriaceae in Southern Thailand. The results reported in this study highlight the critical importance of implementing effective antimicrobial management programs and developing innovative treatment approaches to urgently tackle AMR.

## 1. Introduction

The emergence and spread of antimicrobial resistance (AMR) among Enterobacteriaceae play significant roles to global public health. These bacteria are responsible for a wide range of infections, including urinary tract infections (UTIs), hospital-acquired and ventilator-associated pneumonia (HAP/VAP), complicated intra-abdominal infections (cIAIs), and bloodstream infections (BSIs) [1]. AMR is a critical issue that contributes to significant morbidity and mortality worldwide. According to the World Health Organization (WHO), AMR causes an estimated 700,000 deaths annually, and this number could rise to 10 million by 2050 if no effective measures are taken [2]. The economic burden is also substantial, with healthcare costs rising due to prolonged hospital stays, the need for more expensive treatments, and increased mortality rates. Infections caused by multidrug-resistant (MDR) Enterobacteriaceae are particularly challenging to manage and control, leading to significant treatment costs and posing a threat to public health [3].

In Southern Thailand, where infectious diseases are a major public health concern, understanding the genomic diversity, AMR profiles, and virulence factors of Enterobacteriaceae is crucial for guiding local treatment strategies and infection control measures. Despite the importance of this issue, there is a scarcity of studies focusing on the genomics of Enterobacteriaceae in this region. The ability of this bacterial family to rapidly acquire and disseminate resistance genes has made them difficult challengers in the clinical setting, often resulting in treatment failures and increased healthcare costs.

We obtained a diverse family of bacteria that includes several clinically significant genera, such as *Proteus*, *Serratia*, *Klebsiella*, *Phytobacter*, *Kosakonia*, and *Citrobacter*, from short-term survey of Enterobacteriaceae in Southern Thailand hospitals. Therefore, this study aims to address this gap by providing genomic insights into Enterobacteriaceae diversity, AMR, and virulence in Southern Thailand. By employing whole-genome sequencing and bioinformatic analyses, we seek to characterize the genetic diversity of Enterobacteriaceae isolates, identify AMR genes and mechanisms, and elucidate virulence factors contributing to their pathogenicity. The findings of this study are expected to have significant implications for clinical practice and public health in Southern Thailand. By providing a comprehensive understanding of the genomic landscape of Enterobacteriaceae in this region, we might be able to suggest local treatment guidelines, enhance antimicrobial deployment efforts, and contribute to global AMR surveillance efforts.

## 2. Results and Discussions

### 2.1. Clinical Data and Antimicrobial Susceptibility Testing Results

The clinical data and antimicrobial resistance susceptibility results are exhibited in Table 1, providing a detailed overview of Enterobacteriaceae strains isolated from various hospital sources and their resistance patterns to a range of antibiotics. The included strains are *P. mirabilis*, *S. nevei*, two isolates of *K. variicola*, *K. aerogenes*, *K. indica*, *K. grimontii*, *P. ursingii*, *P. palmae*, *Kosakonia* spp., and two isolates of *C. freundii* from different source of isolations, such as rectal, throat, endotracheal tube, and nasopharynx samples, highlighting the diverse nature of these pathogens and their potential to cause infections in different clinical contexts. All strains in this study present a resistance to carbapenem antibiotics (meropenem, imipenem, and ertapenem) and some types of third-generation cephalosporin (ceftriaxone and ceftazidime), indicating a high level of multidrug resistance among these pathogens. Previous studies have also reported the problem of carbapenem-resistant Enterobacteriaceae (CRE). For example, in 2017, Logan and Weinstein reported on the evolution and epidemiology of CRE globally, highlighting the rapid global dissemination of carbapenemase-producing Enterobacteriaceae [4]. Another study in 2021 emphasized the global challenge of multidrug-resistant (MDR) Enterobacteriaceae and the need for new treatment options to combat CRE infections [5]. In addition to resistance to ß-lactam drugs, the resistance profiles reveal variability among the strains. For instance, *K. variicola* PSU7 shows resistance to most antibiotics except amikacin, while *K. variicola* PSU16 exhibits resistance to all antibiotics tested. This bacterium is recognized as an emerging pathogen in humans and has been identified in various environments. It is a member of the *K. pneumoniae* complex and has been found to be a more serious pathogen, containing a broad range of resistance genes that confer the resistance phenotype. This indicates a particularly worrisome level of MDR, especially the ß-lactam class [5].

**Figure 1:**
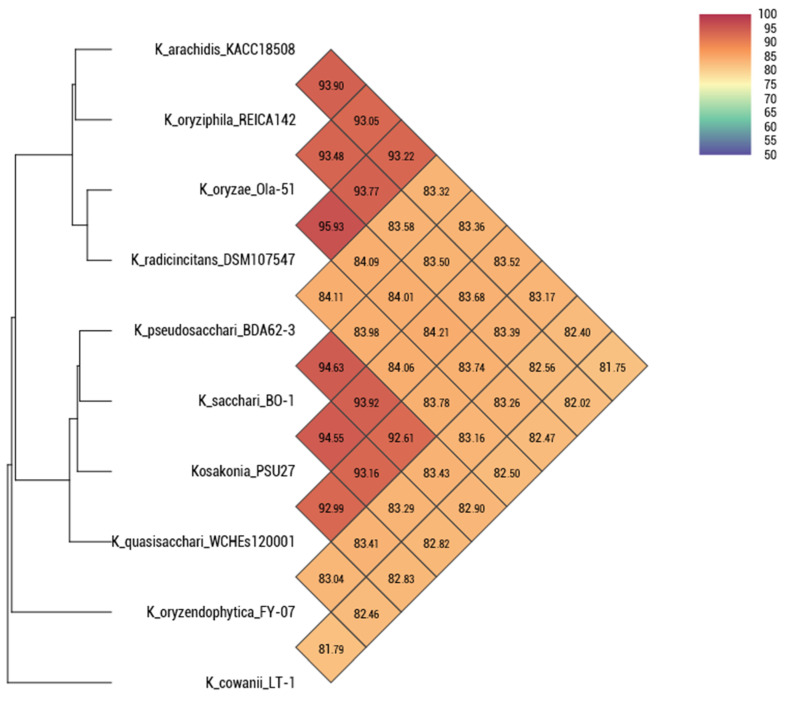
Average nucleotide identity of nine *Kosakonia* isolates collected from RefSeq NCBI database and *Kosakonia* spp. PSU27 in this study. Percent nucleotide identity lower than 95% is considered as a novel species.

Surprisingly, we identified a novel species of *Kosakonia* spp. PSU27, isolated from the nasopharynx of a patient at Satun Hospital. The average nucleotide identity (ANI) shows a 94.55% similarity to the closest species, *Kosakonia sacchari* BO-1. We also collected data from the RefSeq database, which contains 99 isolates of *Kosakonia* genomes (Figure 1). Among these isolates, only five are clinical samples: one *Kosakonia* spp. from an oral metagenome, three *Kosakonia radicincitans* from blood, and one *Kosakonia cawanii* from an oral swab. Our *Kosakonia* spp. PSU27 is the first novel species reported in Thailand.

This discovery of a novel species, *Kosakonia* spp. PSU27, isolated from a patient’s nasopharynx at Satun Hospital, is significant for several reasons. Firstly, it expands the known diversity of the *Kosakonia* genus, which can have implications for understanding its ecological niche, pathogenicity, and potential biotechnological applications. The identification of this novel species was supported by an average nucleotide identity (ANI) analysis, which showed a 94.55% similarity to the closest known species, *Kosakonia sacchari* BO-1. This level of genetic divergence suggests that *Kosakonia* spp. PSU27 represents a distinct species within the genus. Furthermore, the rarity of clinical isolates of *Kosakonia* in public databases, as evidenced by the limited number of clinical samples among the 99 isolates of *Kosakonia* genomes in RefSeq NCBI [6], underscores the novelty of this finding. Among these isolates, only five were clinical samples, which indicates the importance of this discovery in expanding our understanding of the clinical relevance of *Kosakonia* species.

These antimicrobial profiles and the ANI provide valuable insights into the diversity and antimicrobial resistance patterns of Enterobacteriaceae strains in clinical settings. The inclusion of various strains from different hospital sources highlights the diverse nature of these pathogens and their potential to cause infections in different clinical contexts. The high level of multidrug resistance, including resistance to carbapenem antibiotics, highlights the urgent need for new treatment options and effective antimicrobial management programs. The variability in resistance profiles among strains further emphasizes the complexity of antimicrobial resistance mechanisms within Enterobacteriaceae. The discovery of a novel species, *Kosakonia* spp. PSU27, expands our understanding of the genus and raises awareness among clinical staff and researchers about the importance of ongoing surveillance and research to identify emerging pathogens.

### 2.2. Antimicrobial Resistance Genes in Enterobacteriaceae

**Figure 2:**
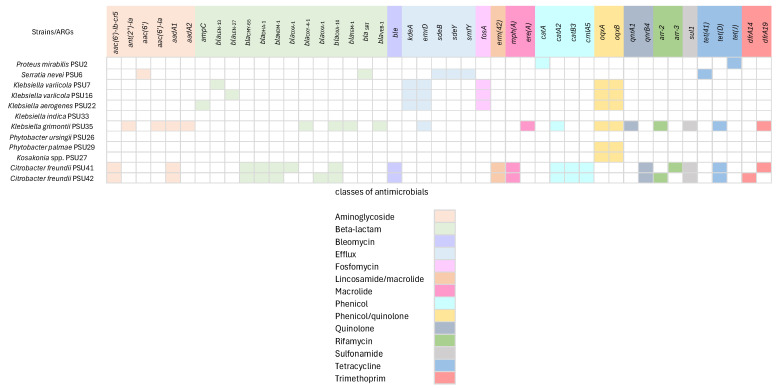
Antimicrobial resistance genes (ARGs) in 13 Enterobacteriaceae isolates. Forty-five genes conferring antimicrobial resistance genes which were categorized into 14 classes. The presence of the genes is represented by different colors. Classes of related antimicrobial resistance genes are represented below.

The Enterobacteriaceae isolates in this study exhibit a wide range of antimicrobial resistance genes, with a total of 45 ARGs, which were categorized into 14 classes based on the antibiotics they confer resistance to (Figure 2). Among the isolates, *Klebsiella* and *Citrobacter* strains were found to carry several ß-lactamase genes (e.g., *bla*_LEN_, *bla*_CMY_, *bla*_DHA_, *bla*_NDM_, *bla*_OXA_, *bla*_TEM_, and *bla*_VEB_), indicating resistance to ß-lactam antibiotics. The presence of these genes suggests the enzymatic inactivation of beta-lactams. A study by Chen et al. found that *bla*_TEM_, *bla*_CTX-M_, and *bla*_OXA_ were the most common ß-lactamase genes detected in Enterobacteriaceae isolates [7], aligning with our findings. Furthermore, our study identified additional resistance genes, including those for efflux pumps (e.g., *kdeA*, *emrD*, *sdeB*, *sdeY*, and *smfY*) in strains such as *S. nevei* PSU6 and some isolates of *Klebsiella*. Efflux pumps, regulated by these genes, play a crucial role in bacterial survival by expelling toxic substances, including antibiotics. Wang et al. similarly identified efflux pump genes, such as *acrAB* and *oqxAB*, in Enterobacteriaceae isolates, which contribute significantly to multidrug resistance [8].

For Fosfomycin resistance (*fosA*) gene detection, studies from Hong Kong and Japan have examined the prevalence of fosfomycin resistance in clinical isolates of Enterobacteriaceae, including *Klebsiella* species. These studies revealed that the *fosA* gene is commonly found among resistant isolates, indicating a widespread distribution of this resistance determinant. For example, a study in Hong Kong reported a high prevalence of *fosA* among fosfomycin-resistant clinical isolates, emphasizing the significance of this gene in conferring resistance [9]. Likewise, research from Japan identified *fosA* as a frequent contributor to fosfomycin resistance in Enterobacteriaceae [9]. The presence of *fosA* in our *Klebsiella* isolates aligns with these findings and suggests the gene’s role in resistance mechanisms and its potential impact on the efficacy of fosfomycin as a treatment option. This highlights the importance of monitoring the prevalence of *fosA* and other resistance genes to inform treatment strategies and combat antibiotic resistance. Additionally, genes associated with phenicol resistance (*catA* and *catB*), quinolone resistance (*oqxA* and *oqxB*), sulfonamide resistance (*sul1*), and tetracycline resistance (*tet(41)*, *tet(D)* and *tet(J)*) were found in various strains. These identified ARGs are mostly related to AST results, indicating the presence of these genes correlates with observed resistance phenotypes.

There are some discrepancies observed in *P. ursingii* PSU26, which shows no ARGs, and *P. palmae* PSU29 and *Kosakonia* spp. PSU27, which contain only *oqxA* and *oqxB*. However, this species was shown to resist all tested antimicrobials. This could be explained by the capability of the efflux system regulated by *oqxAB* [10]. The absence of ARGs in *P. ursingii* PSU26 and *K. indica* PSU33 is particularly important and could suggest alternative mechanisms of resistance. One such mechanism is the presence of multidrug efflux systems, which are known to confer resistance by actively pumping out antibiotics from the bacterial cell, thus reducing their intracellular concentrations to sub-lethal levels [11]. Another possible mechanism could be modifications in the bacterial cell membrane permeability. Bacteria can alter their outer membrane porins, reducing antibiotic uptake, and, thereby, conferring resistance. Additionally, the enzymatic inactivation of antibiotics through less well-characterized mechanisms or the presence of chromosomal mutations that confer resistance could also play roles [12]. Furthermore, short-read sequencing tends to produce errors and limitations that may impact the accuracy of results, especially in complex genomic regions or when dealing with highly similar sequences [13]. In addition, the presence of only *oqxA* and *oqxB* in *P. palmae* PSU29 and *Kosakonia* spp. PSU27 raises questions about the mechanisms underlying their MDR. These isolates might rely on specific efflux pumps, encoded by these genes, to expel multiple antibiotics from their cells [14]. The results suggest the need for effective antibiotic management programs and the development of novel antimicrobial agents to combat infections caused by MDR bacteria.

### 2.3. Stress Response, Metal Tolerance, and Virulence-Associated Genes in Enterobacteriaceae

**Figure 3:**
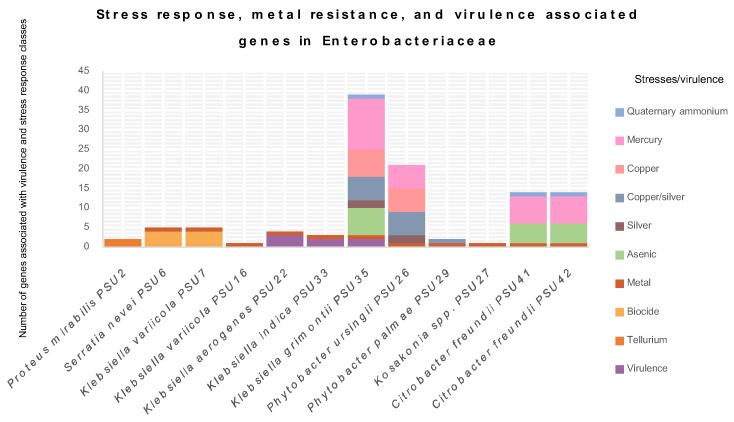
The graph depicts the determination of stress response, metal tolerance, and virulence-associated genes in 12 Enterobacteriaceae isolates from this study. Different colors represent classes of stress or virulence.

A comprehensive examination of stress response, metal tolerance, and virulence genes in Enterobacteriaceae was presented in Figure 3. This study targeted these specific genes because they play crucial roles in the survival and pathogenicity of bacteria, impacting both human health and environmental management. Stress response genes help bacteria adapt to various environmental challenges, including oxidative stress, osmotic stress, and heat shock, which are common in hospital and natural settings. These genes are involved in the cellular response to environmental stressors and are essential for bacterial survival in challenging conditions [15]. Metal tolerance genes are vital due to the increasing environmental contamination with metals, which not only affects bacterial survival but can also be linked to antibiotic resistance mechanisms, posing significant treatment challenges. Virulence genes are essential for understanding the pathogenic potential, as they contribute to the ability of bacteria to cause disease in hosts.

The results reveal that all Enterobacteriaceae in this study contain at least one type of virulence or stress gene. *P. mirabilis* PSU2 possesses the *terD* and *terZ* genes, conferring tellurium resistance. *S. nevei* PSU6 exhibits biocide-regulating genes, such as *smdA*, *smdB*, *sdeA*, and *ssmE*, associated with multidrug efflux systems. These systems play a crucial role in bacterial resistance to antimicrobial compounds, including antibiotics, by pumping them out of the cell [16,17,18]. The strain PSU6 also carries *fieF*, which confers metal tolerance. This gene is identified in almost all isolates in this study, except for *P. mirabilis* PSU2. Generally, Enterobacteriaceae can exhibit intrinsic or acquired resistance mechanisms, often involving efflux pumps or metal-binding proteins [19]. Metal tolerance is concerning, as it can be related to antibiotic resistance, potentially leading to treatment challenges [20]. Moreover, environmental contamination with metals can further contribute to the development and spread of metal resistance in this family.

Other interesting virulence-associated genes detected were the *iroB*, *iroC*, and *iroN* genes, carried by *K. aerogenes* PSU22. These genes are important components of the iron acquisition system in *Escherichia coli*, related to the production, export, and uptake of the siderophore salmochelin, crucial for scavenging iron, an essential nutrient, particularly in environments with limited iron [21]. For *ybtP*, *ybtQ*, and the yersiniabactin ABC Transporter ATP-Binding/Permease Protein, detected in *K. indica* PSU33 and *K. grimontii* PSU35, they are key components of the yersiniabactin gene cluster in Enterobacteriaceae, contributing to the biosynthesis and uptake of the siderophore yersiniabactin, crucial for acquiring iron from the host environment [22]. The details of stress response, metal tolerance, and virulence-associated genes are displayed in Table S2. The presence of multidrug efflux genes in *S. nevei* PSU6 highlights its ability to resist various antimicrobial agents, which might explain the lack of sensitivity to all tested antimicrobials in this isolate. *K. variicola* PSU7 and *P. mirabilis* PSU2 exhibit genes for tellurite resistance, which is particularly interesting given the scarceness of tellurium resistance mechanisms in bacteria [23]. *K. aerogenes* PSU22 and *K. indica* PSU33 display genes associated with virulence, indicating their potential pathogenicity. *K. grimontii* PSU35 and PSU36 are notable for their extensive repertoire of stress response genes, suggesting their adaptability to diverse environmental stresses, including metals, arsenic, and biocides [24,25]. *P. ursingii* PSU26 and *P. palmae* PSU29 also exhibit stress response genes, highlighting their ability to survive in metal-contaminated environments. *Kosakonia* spp. PSU27 demonstrates a similar trend, indicating its adaptation to metal stress. *C. freundii* PSU41 and PSU42 show a combination of stress response genes related to metals, arsenic, and mercury, along with genes conferring resistance to quaternary ammonium compounds, reflecting their flexibility against multiple stressors [26]. Understanding these genes emphasizes the genetic diversity and adaptive capacity of bacteria, offering insights into their survival strategies and potential implications for human health and environmental management.

### 2.4. Plasmid Identification in Enterobacteriaceae

**Figure 4:**
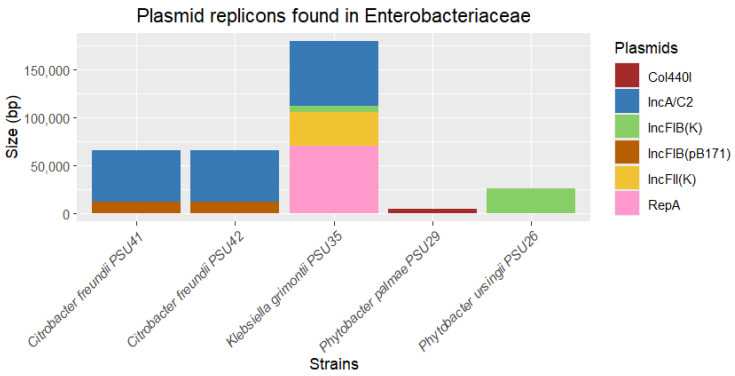
Determination of plasmid replicons identified from five out of twelve Enterobacteriaceae in this study. The thickness of each stack indicates the size of sequences, and different colors represent the types of replicons.

According to PlasmidFinder, various plasmid replicons were found in bacterial strains, along with their lengths. In this study, plasmid replicons were identified in five isolates: *P. ursingii* PSU26, *P. palmae* PSU29, *K. grimontii* PSU35, and *C. freundii* PSU42 and PSU41. The identified plasmids included IncFIB(K), Col440I, IncA/C2, IncFIB(pB171), IncFII(K), and RepA, ranging in length from 4332 to 70,146 base pairs (Figure 4). The most abundant plasmid replicon type found was IncA/C2, identified in *C. freundii* PSU41 and PSU42, and *K. grimontii* PSU35 (Figure 4). IncA/C2 is commonly found in Enterobacteriaceae and other Gram-negative bacteria, known for carrying multiple ARGs and other mobile genetic elements [27,28].

A further analysis using the PLSDB tool confirmed that these isolates carry plasmids with a high sequence identity to those found in a variety of bacterial species globally. A PLSDB analysis identified several plasmids in Enterobacteriaceae isolates. These plasmids range from 1657 to 71,960 base pairs and are associated with strains from diverse geographic locations, including China, the USA, Canada, the UK, India, Japan, and Hong Kong. The same set of plasmids was identified in two isolates of *C. freundii*. These identified plasmids are associated with a variety of bacterial species, such as *Enterobacter hormaechei*, *Citrobacter* sp., *Salmonella enterica*, *Klebsiella pneumoniae*, *Raoultella* sp., *Klebsiella quasipneumoniae*, and *Enterobacter kobei* (Table 2). The presence of high-sequence-identity plasmids in these isolates probably suggests a potential for horizontal gene transfer and the global dissemination of plasmid-borne genes [29]. This is particularly significant for genes related to antimicrobial resistance and virulence, highlighting the role of plasmids in spreading resistance traits across different bacterial species and geographic regions. Moreover, the identical set of plasmids present in *C. freundii* collected from different samples might indicate a clonal spread or a common source of infection, suggesting that these plasmids confer a selective advantage that allows for their persistence and proliferation within and across different hosts [30]. The varying lengths of plasmid sequences underscore the diverse nature of plasmid genomes, which can carry different sets of genes that contribute to bacterial adaptability and survival. Taxonomically, the plasmids identified in this study are associated with several clinically significant bacterial species. Specially, species such as *K. pneumoniae* and *S. enterica* are known for their roles in hospital-acquired infections and their ability to acquire multidrug resistance [31].

Despite identifying several ARGs and plasmid, no ARGs were found to be mediated by the plasmids. This could be because ARGs may be located on the bacterial chromosome rather than on plasmids. Furthermore, the ARGs may have integrated into the bacterial chromosome from plasmids through mechanisms such as transposition or recombination. The short-read sequencing used in this study could not obtain the complete genome of the bacteria, leading to limitations in identifying plasmid–ARG associations, such as sensitivity issues or incomplete plasmid databases [32,33]. The detection of these plasmids in our isolates might suggest that this study may uncover critical information regarding the spread and characteristics of resistance plasmids in different bacterial hosts.

## 3. Materials and Methods

### 3.1. Sample Collection

This study is part of a short-term surveillance effort conducted in ICU patients from five different hospitals in Southern Thailand over a 6-month period in 2019 [34,35,36]. The project identified 241 Enterobacteriaceae suspected of being resistant to carbapenem antimicrobials, all screened for resistance using MacConkey agar supplemented with 2 µg/mL of imipenem. Twelve out of 241 Enterobacteriaceae isolates were selected for this study. These included one *Proteus mirabilis*, one *Serratia nevei*, two *Klebsiella variicola*, one *Klebsiella aerogenes*, one *Klebsiella indica*, one *Klebsiella grimontii*, one *Phytobacter ursingii*, one *Phytobacter palmae*, one *Kosakonia* spp., and two *Citrobacter freundii*. They were chosen because they are uncommon in clinical samples but are found in outside communities or contaminated surfaces in the environment. These twelve isolates were collected from patients admitted to intensive care units (ICUs) in hospitals in southern Thailand, including Songklanagarind Hospital, Patthalung Hospital, Satun Hospital, Pattani Hospital, and Yala Hospital. The bacteria were isolated from various specimens, such as nasopharynx, swabs, endotracheal tubes, throats, and rectums. All patients had received prior antibiotics before their specimens were collected (Table S1).

Initial species identification of the twelve strains was initially conducted using biochemical tests based on Bergey’s Manual of Systematic Bacteriology [37], with further confirmation accomplished using Matrix-Assisted Laser Desorption/Ionization–Time of Flight (MALDI-TOF) mass spectrometry (MS). However, recognizing the limitations of MALDI-TOF MS in distinguishing between species within the *Klebsiella pneumoniae* complex, we confirmed the identification of our selected. All of them were stored at −20 °C for further identification and analysis using WGS.

### 3.2. Antimicrobial Susceptibility Testing

All isolates underwent antimicrobial susceptibility testing (AST) using the disk diffusion method, according to Kirby–Bauer disk diffusion susceptibility test protocol [38]. The study utilized antimicrobial disks, including ciprofloxacin (5 µg), levofloxacin (5 µg), amikacin (10 µg), gentamicin (10 µg), imipenem (10 µg), meropenem (10 µg), tazocin (100/10 µg), ertapenem (30 µg), ceftriaxone (30 µg), ceftazidime (30 µg), and sulperazon (100/10 µg). *Escherichia coli* ATCC^®^ 25922 (for co-trimoxazole) and *Pseudomonas aeruginosa* ATCC^®^ 27853 were used as quality controls. AST results were interpreted according to the Clinical & Laboratory Standards Institute (CLSI) standard [39].

### 3.3. DNA Extraction and Sequencing

The genomic DNA from all Enterobacteriaceae isolates were obtained by the TIANamp Bacterial DNA Kit (Tiangen, Beijing, China), followed the manufacturer’s guidelines [40]. The DNA concentrations were assessed using a NanoDrop™ 2000/2000c Spectrophotometer (Thermo Scientific, Norristown, PA, USA), while the integrity and purity of the DNA were verified through Agarose Gel Electrophoresis. Subsequently, the DNA samples were sent to the Beijing Genomics Institute (BGI) for short-read WGS with 150 bp paired-end reads using MGISEG-2000 platform.

### 3.4. Bioinformatics and Sequence Analysis

The BacSeq pipeline (Accession date: 18 February 2024) [41], a program-based bioinformatic tool for analyzing bacterial genomes, was used for processing Enterobacteriaceae genomes. All analyses and programs used in this study were performed using default parameters. Firstly, FastQC was utilized for quality control of the sequences [42]. The sequences that passed quality control were then assembled using SPAdes [43]. The assembly quality and completeness were checked using Quast and BUSCO [44,45], respectively. Genome annotation was performed using Prokka [46], and all annotated results were employed for downstream analysis. For the downstream analysis, AMRFinderPlus (Accession date: 1 April 2024) [47] was utilized for the detection of antimicrobial resistance, stress response, and virulence genes, while plasmid replicon and plasmid were identified using PlasmidFinder (Accession date: 6 April 2024) and PLSDB (Accession date: 20 May 2024) [48,49]. RStudio version 4.3.2 (Accession date: 22 April 2024) was used for generating figures [50]. To confirm the novel species of *Kosakonia* spp. PSU27, OrthoANI (Accession date: 12 April 2024) [51] was used to evaluate average nucleotide identity (ANI) of *Kosakonia* spp. PSU27 and other nine representative clinical isolates of *Kosakonia* obtained from RefSeq NCBI. An identity value lower than 95% is considered indicative of a novel species. The complete plasmid was selected for comparison with highly similar plasmids available in the NCBI database and was visualized using Proksee (Accession date: 21 April 2024) [52].

## 4. Conclusions

This study provides valuable insights into the genomic characteristics, antimicrobial resistance profiles, and virulence factors of Enterobacteriaceae strains isolated from hospitals in Southern Thailand. The findings highlight the diversity and complexity of these pathogens, emphasizing their potential to cause a wide range of infections and the challenges they pose to clinical management. The high prevalence of multidrug resistance, particularly to carbapenem antibiotics, is a major concern and underscores the urgent need for effective antimicrobial management programs and the development of new treatment options. The variability in resistance profiles among strains further emphasizes the need for tailored treatment strategies based on accurate identification and susceptibility testing. This necessitates the regular monitoring of resistance patterns and the implementation of precise diagnostic methods, such as whole-genome sequencing, to guide the appropriate therapy. The discovery of a novel species, *Kosakonia* spp. PSU27, expands our understanding of the genus and highlights the importance of ongoing surveillance for emerging pathogens. Moreover, the presence of various stress response, metal tolerance, and virulence genes underscores the adaptability and pathogenic potential of these bacteria. These genetic traits contribute to their survival in harsh environments and their ability to evade host immune responses, posing significant challenges in both clinical and environmental settings.

Furthermore, we identified plasmids with a high sequence identity to those found in various bacterial species globally, highlighting the extensive horizontal gene transfer occurring across diverse geographic locations and bacterial species. The identification of multiple plasmids in isolates from this study, which match those detected in other regions, emphasizes the global challenge posed by plasmid-mediated resistance. This finding points to the need for robust monitoring systems and effective strategies to limit the dissemination of resistance genes within microbial populations. Our study underscores the critical need for continuous surveillance and increased awareness among healthcare staff regarding these rare, high-virulence, and multidrug-resistant pathogens. Understanding the local epidemiology of these pathogens is vital for informing regional public health strategies and improving infection control practices. The data collected from hospitals in Southern Thailand provide a representative snapshot of the current challenges faced in this area, highlighting the necessity for an enhanced healthcare infrastructure to better manage and mitigate the risks posed by these dangerous pathogens.

Further research is needed to elucidate the mechanisms underlying resistance and virulence in these strains. This includes studying the regulation and expression of resistance and virulence genes, as well as investigating the ecological and evolutionary pressures that drive their emergence and persistence. Understanding these mechanisms will be crucial for developing innovative strategies to combat the spread of multidrug-resistant Enterobacteriaceae.

## Figures and Tables

**Table 1:** Antimicrobial susceptibility testing in Enterobacteriaceae strains in this study.

Strain	Hospital	Source	Antimicrobial Susceptibility Test (AST)										
			MEM	IPM	ETP	GEN	AMK	TZP	CIP	LVX	CRO	CAZ	SAM
*Proteus mirabilis* PSU2	PSU	R	R	R	R	S	R	R	R	R	R	R	R
*Serratia nevei* PSU6	PSU	Th	R	R	R	R	R	R	I	I	R	R	R
*Klebsiella variicola* PSU7	PSU	R	R	R	R	R	S	R	R	R	R	R	R
*Klebsiella variicola* PSU16	PT	R	R	R	R	R	R	R	R	R	R	R	R
*Klebsiella aerogenes* PSU22	ST	Tu	R	R	R	R	S	R	R	R	R	R	R
*Klebsiella indica* PSU33	PA	Th	R	R	R	S	R	R	R	R	R	R	S
*Klebsiella grimontii* PSU35	PA	Ng	R	R	R	R	R	R	R	R	R	R	R
*Phytobacter ursingii* PSU26	ST	Ng	R	R	R	R	R	R	I	R	R	R	R
*Phytobacter palmae* PSU29	ST	Ng	R	R	R	S	R	R	R	I	R	R	R
*Kosakonia* spp. PSU27	ST	Ng	R	R	R	R	R	R	R	R	R	R	R
*Citrobacter freundii* PSU41	YL	R	R	R	R	R	S	R	I	R	R	R	S
*Citrobacter freundii* PSU42	YL	R	R	R	R	R	R	R	R	R	R	R	R
Hospital							Source of isolation						
PSU: Songklanagarind Hospital							Ng: nasopharynx						
PT: Patthalung Hospital							Tu: endotracheal tube						
ST: Satun Hospital							Th: throat						
PA: Pattani Hospital							R: rectum						
YL: Yala Hospital													

Meropenem (MEM), Imipenem (IPM), Ertapenem (ETP), Gentamicin (GEN), Amikacin (AMK), Tazocin (TZP), Ciprofloxacin (CIP), Levofloxacin (LVX), Ceftriaxone (CRO), Ceftazidime (CAZ), Sulperazon (SAM). Resistance (R), Susceptible (S), and Intermediate (I). The results were analyzed according to the Clinical and Laboratory Standards Institute 2023 (CLSI 2023).

**Table 2:** Plasmid identification using PLSDB.

Isolate	Accession No.	Identity	Length	Location	Taxonomy
*Phytobacter ursingii* PSU26	NZ_CP083851.1	0.998551	3115	China	*Enterobacter hormaechei*
*Phytobacter palmae* PSU29	NZ_CP056253.1	0.98551	71,960	United Kingdom	*Citrobacter* sp. RHBSTW-00903
	CP101347.1	0.984609	1702	China	*Salmonella enterica*
	CP093099.1	0.983292	4448	USA	*Salmonella enterica*
	NZ_CP038598.1	0.983159	4448	Canada	*Salmonella enterica*
	CP093106.1	0.982958	4448	USA	*Salmonella enterica*
	CP093079.1	0.982958	4448	USA	*Salmonella enterica*
	NZ_CP113909.1	0.982282	4448	United Kingdom	*Klebsiella michiganensis*
*Klebsiella grimontii* PSU35	NZ_CP079817.1	1	1657	India	*Klebsiella pneumoniae*
	NZ_CP093279.1	0.999377	20,796	China	*Raoultella* sp. HC6
	NZ_AP026417.1	0.999038	2889	Japan	*Klebsiella quasipneumoniae*
*Citrobacter freundii* PSU41	NZ_MN370929.1	1	3579	missing	*Klebsiella pneumoniae*
	NZ_CP079817.1	1	1657	India	*Klebsiella pneumoniae*
	NZ_CP079667.1	0.999522	2186	India	*Klebsiella pneumoniae*
	NZ_CP083865.1	0.999473	17,041	China	*Enterobacter kobei*
	NZ_CP069298.1	0.999425	17,017	China	*Salmonella enterica*
	NZ_CP055215.1	0.999281	13,247	Hong Kong	*Klebsiella quasipneumoniae*
*Citrobacter freundii* PSU42	NZ_CP079817.1	1	1657	India	*Klebsiella pneumoniae*
	NZ_MN370929.1	0.999618	3579	missing	*Klebsiella pneumoniae*
	NZ_CP079667.1	0.999522	2186	India	*Klebsiella pneumoniae*
	NZ_CP083865.1	0.999425	17,041	China	*Enterobacter kobei*
	NZ_CP069298.1	0.999377	17,017	China	*Salmonella enterica*
	NZ_CP055215.1	0.999184	13,247	Hong Kong	*Klebsiella quasipneumoniae*

## Data Availability

The assembled genomes of all Enterobacteriaceae isolates in this study have been deposited in the NCBI GenBank under BioProject number PRJNA1080727, with BioSample numbers SAMN40146458 to SAMN40146472.

## Supplementary Materials

The following supporting information can be downloaded at: https://www.mdpi.com/article/10.3390/antibiotics13060531/s1, Table S1: Demographic data of isolates in this study. Table S2: Stress response, metal resistance, and virulence-associated genes.
