# Supplementary material for: Genomic Characterization of Multidrug-Resistant Enterobacteriaceae Clinical Isolates from Southern Thailand Hospitals: Unraveling Antimicrobial Resistance and Virulence Mechanisms
Source: Antibiotics (Basel). 2024 Jun 6;13(6):531. doi: 10.3390/antibiotics13060531 (PMC11200480; doi:10.3390/antibiotics13060531)
Supplement: Supplementary file 1 [file antibiotics-13-00531-s001.zip › antibiotics-3012073-supplementary Table S2.pdf]

Supplementary Table S2. stress response, metal resistance, and virulence-associated genes

Name	Gene	Sequence Name	Scope	Type	Class
***Proteus mirabilis* PSU2**	*terD*	Tellurium Resistance Membrane Protein Terd	Stress	Metal	Tellurium
	terZ	Tellurium Resistance-Associated Protein Terz	Stress	Metal	Tellurium
***Serratia nevei* PSU6**	*smdA*	Multidrug Efflux ABC Transporter Permease/ATP-Binding Subunit Smda	Stress	Biocide	Efflux
	smdB	Multidrug Efflux ABC Transporter Permease/ATP-Binding Subunit Smdb	Stress	Biocide	Efflux
	sdeA	Multidrug Efflux RND Transporter Periplasmic Adaptor Subunit Sdea	Stress	Biocide	Efflux
	ssmE	Multidrug Efflux SMR Transporter Ssme	Stress	Biocide	Efflux
	fieF	CDF Family Cation-Efflux Transporter Fief	Stress	Metal	Na
***Klebsiella variicola* PSU7**	*fieF*	CDF Family Cation-Efflux Transporter Fief	Stress	Metal	Na
	terE	Tellurium Resistance Camp Binding Protein Tere	Stress	Metal	Tellurium
	terD	Tellurium Resistance Membrane Protein Terd	Stress	Metal	Tellurium
	terC	Tellurium Resistance Membrane Protein Terc	Stress	Metal	Tellurium
	terB	Tellurium Resistance Membrane Protein Terb	Stress	Metal	Tellurium
***Klebsiella variicola* PSU16**	*fieF*	CDF Family Cation-Efflux Transporter Fief	Stress	Metal	Na

Supplementary Table S2. stress response, metal resistance, and virulence-associated genes

Name	Gene	Sequence Name	Scope	Type	Class
***Klebsiella aerogenes* PSU22**	*iroB*	Salmochelin Biosynthesis C-Glycosyltransferase Irob	Virulence	Virulence	Na
	iroC	Salmochelin/Enterobactin Export ABC Transporter Iroc	Virulence	Virulence	Na
	iroN	Siderophore Salmochelin Receptor Iron	Virulence	Virulence	Na
	fieF	CDF Family Cation-Efflux Transporter Fief	Stress	Metal	Na
***Klebsiella indica* PSU33**	*ybtP*	Yersiniabactin ABC Transporter ATP-Binding/Permease Protein Ybtp	Virulence	Virulence	Na
	ybtQ	Yersiniabactin ABC Transporter ATP-Binding/Permease Protein Ybtq	Virulence	Virulence	Na
	fieF	CDF Family Cation-Efflux Transporter Fief	Stress	Metal	Na
***Klebsiella grimontii* PSU35**	*ybtQ*	Yersiniabactin ABC Transporter ATP-Binding/Permease Protein Ybtq	Virulence	Virulence	Na
	ybtP	Yersiniabactin ABC Transporter ATP-Binding/Permease Protein Ybtp	Virulence	Virulence	Na
	arsC	Glutaredoxin-Dependent Arsenate Reductase	Stress	Metal	Arsenate
	arsB	Arsenite Efflux Transporter Membrane Subunit Arsb	Stress	Metal	Arsenite
	fieF	CDF Family Cation-Efflux Transporter Fief	Stress	Metal	Na
	silE	Silver-Binding Protein Sile	Stress	Metal	Silver
	silS	Copper/Silver Sensor Histidine Kinase Sils	Stress	Metal	Copper/Silver

Supplementary Table S2. stress response, metal resistance, and virulence-associated genes

Name	Gene	Sequence Name	Scope	Type	Class
***Klebsiella grimontii* PSU35**	*silR*	Copper/Silver Response Regulator Transcription Factor Silr	Stress	Metal	Copper/Silver
	silC	Cu(+)/Ag(+) Efflux RND Transporter Outer Membrane Channel Silc	Stress	Metal	Copper/Silver
	silF	Cu(+)/Ag(+) Efflux RND Transporter Periplasmic Metallochaperone Silf	Stress	Metal	Copper/Silver
	silB	Cu(+)/Ag(+) Efflux RND Transporter Periplasmic Adaptor Subunit Silb	Stress	Metal	Copper/Silver
	silA	Cu(+)/Ag(+) Efflux RND Transporter Permease Subunit Sila	Stress	Metal	Copper/Silver
	silP	Ag(+)-Translocating P-Type Atpase Silp	Stress	Metal	Silver
	pcoA	Multicopper Oxidase Pcoa	Stress	Metal	Copper
	pcoB	Copper-Binding Protein Pcob	Stress	Metal	Copper
	pcoC	Copper Resistance System Metallochaperone Pcoc	Stress	Metal	Copper
	pcoD	Copper Resistance Inner Membrane Protein Pcod	Stress	Metal	Copper
	pcoR	Copper Response Regulator Transcription Factor Pcor	Stress	Metal	Copper
	pcoS	Copper Resistance Membrane Spanning Protein Pcos	Stress	Metal	Copper
	pcoE	Copper Resistance System Metallochaperone Pcoe	Stress	Metal	Copper
	arsC	Glutaredoxin-Dependent Arsenate Reductase	Stress	Metal	Arsenate
	arsB	Arsenite Efflux Transporter Membrane Subunit Arsb	Stress	Metal	Arsenite
	arsA	Arsenite Efflux Transporter Atpase Subunit Arsa	Stress	Metal	Arsenite

Supplementary Table S2. stress response, metal resistance, and virulence-associated genes

Name	Gene	Sequence Name	Scope	Type	Class
***Klebsiella grimontii* PSU35**	*arsD*	Arsenite Efflux Transporter Metallochaperone Arsd	Stress	Metal	Arsenite
	arsR	As(III)-Sensing Metalloregulatory Transcriptional Repressor Arsr	Stress	Metal	Arsenic
	merR	Mercury Resistance Transcriptional Regulator Merr	Stress	Metal	Mercury
	merT	Mercuric Transport Protein Mert	Stress	Metal	Mercury
	merP	Mercury Resistance System Periplasmic Binding Protein Merp	Stress	Metal	Mercury
	merF	Mercury Resistance System Transport Protein Merf	Stress	Metal	Mercury
	merD	Mercury Resistance Co-Regulator Merd	Stress	Metal	Mercury
	merE	Broad-Spectrum Mercury Transporter Mere	Stress	Metal	Mercury
	merE	Broad-Spectrum Mercury Transporter Mere	Stress	Metal	Mercury
	merD	Mercury Resistance Co-Regulator Merd	Stress	Metal	Mercury
	merB	Organomercurial Lyase Merb	Stress	Metal	Organomercury
	merA	Mercury(II) Reductase	Stress	Metal	Mercury
	merP	Mercury Resistance System Periplasmic Binding Protein Merp	Stress	Metal	Mercury
	merT	Mercuric Transport Protein Mert	Stress	Metal	Mercury
	merR	Mercury Resistance Transcriptional Regulator Merr	Stress	Metal	Mercury

Supplementary Table S2. stress response, metal resistance, and virulence-associated genes

Name	Gene	Sequence Name	Scope	Type	Class
***Klebsiella grimontii* PSU35**	*qacL*	Quaternary Ammonium Compound Efflux SMR Transporter Qacl	Stress	Biocide	Quaternary Ammonium
Phytobacter ursingii PSU26	*fieF*	CDF Family Cation-Efflux Transporter Fief	Stress	Metal	Na
	merR	Mercury Resistance Transcriptional Regulator Merr	Stress	Metal	Mercury
	merT	Mercuric Transport Protein Mert	Stress	Metal	Mercury
	merP	Mercury Resistance System Periplasmic Binding Protein Merp	Stress	Metal	Mercury
	merF	Mercury Resistance System Transport Protein Merf	Stress	Metal	Mercury
	merD	Mercury Resistance Co-Regulator Merd	Stress	Metal	Mercury
	merE	Broad-Spectrum Mercury Transporter Mere	Stress	Metal	Mercury
	pcoS	Copper Resistance Membrane Spanning Protein Pcos	Stress	Metal	Copper
	pcoR	Copper Response Regulator Transcription Factor Pcor	Stress	Metal	Copper
	pcoD	Copper Resistance Inner Membrane Protein Pcod	Stress	Metal	Copper
	pcoC	Copper Resistance System Metallochaperone Pcoc	Stress	Metal	Copper

Supplementary Table S2. stress response, metal resistance, and virulence-associated genes.

Name	Gene	Sequence Name	Scope	Type	Class
Phytobacter ursingii PSU26	*pcoB*	Copper-Binding Protein Pcob	Stress	Metal	Copper
	pcoA	Multicopper Oxidase Pcoa	Stress	Metal	Copper
	silP	Ag(+)-Translocating P-Type Atpase Silp	Stress	Metal	Silver
	silA	Cu(+)/Ag(+) Efflux RND Transporter Permease Subunit Sila	Stress	Metal	Copper/Silver
	silB	Cu(+)/Ag(+) Efflux RND Transporter Periplasmic Adaptor Subunit Silb	Stress	Metal	Copper/Silver
	silF	Cu(+)/Ag(+) Efflux RND Transporter Periplasmic Metallochaperone Silf	Stress	Metal	Copper/Silver
	silC	Cu(+)/Ag(+) Efflux RND Transporter Outer Membrane Channel Silc	Stress	Metal	Copper/Silver
	silR	Copper/Silver Response Regulator Transcription Factor Silr	Stress	Metal	Copper/Silver
	silS	Copper/Silver Sensor Histidine Kinase Sils	Stress	Metal	Copper/Silver
	silE	Silver-Binding Protein Sile	Stress	Metal	Silver
***Phytobacter palmae* PSU29**	*fieF*	CDF Family Cation-Efflux Transporter Fief	Stress	Metal	Na
	silA	Cu(+)/Ag(+) Efflux RND Transporter Permease Subunit Sila	Stress	Metal	Copper/Silver

Supplementary Table S2. stress response, metal resistance, and virulence-associated genes.

Name	Gene	Sequence Name	Scope	Type	Class
***Kosakonia* spp. PSU27**	*fieF*	CDF Family Cation-Efflux Transporter Fief	Stress	Metal	Na
***Citrobacter freundii* PSU41**	*fieF*	CDF Family Cation-Efflux Transporter Fief	Stress	Stress	Na
	arsC	Glutaredoxin-Dependent Arsenate Reductase	Stress	Stress	Arsenic
	arsB	Arsenite Efflux Transporter Membrane Subunit Arsb	Stress	Stress	Arsenic
	arsA	Arsenite Efflux Transporter Atpase Subunit Arsa	Stress	Stress	Arsenic
	arsD	Arsenite Efflux Transporter Metallochaperone Arsd	Stress	Stress	Arsenic
	arsR	As(III)-Sensing Metalloregulatory Transcriptional Repressor Arsr	Stress	Stress	Arsenic
	merE	Broad-Spectrum Mercury Transporter Mere	Stress	Stress	Mercury
	merD	Mercury Resistance Co-Regulator Merd	Stress	Stress	Mercury
	merB	Organomercurial Lyase Merb	Stress	Stress	Mercury
	merA	Mercury(II) Reductase	Stress	Stress	Mercury
	merP	Mercury Resistance System Periplasmic Binding Protein Merp	Stress	Stress	Mercury
	merT	Mercuric Transport Protein Mert	Stress	Stress	Mercury
	merR	Mercury Resistance Transcriptional Regulator Merr	Stress	Stress	Mercury
	qacL	Quaternary Ammonium Compound Efflux SMR Transporter Qacl	Stress	Stress	Quaternary Ammonium

Supplementary Table S2. stress response, metal resistance, and virulence-associated genes

Name	Gene	Sequence Name	Scope	Type	Class
***Citrobacter freundii* PSU42**	*fieF*	CDF Family Cation-Efflux Transporter Fief	Stress	Metal	Na
	arsC	Glutaredoxin-Dependent Arsenate Reductase	Stress	Metal	Arsenate
	arsB	Arsenite Efflux Transporter Membrane Subunit Arsb	Stress	Metal	Arsenite
	arsA	Arsenite Efflux Transporter Atpase Subunit Arsa	Stress	Metal	Arsenite
	arsD	Arsenite Efflux Transporter Metallochaperone Arsd	Stress	Metal	Arsenite
	arsR	As(III)-Sensing Metalloregulatory Transcriptional Repressor Arsr	Stress	Metal	Arsenic
	merE	Broad-Spectrum Mercury Transporter Mere	Stress	Metal	Mercury
	merD	Mercury Resistance Co-Regulator Merd	Stress	Metal	Mercury
	merB	Organomercurial Lyase Merb	Stress	Metal	Organomercury
	merA	Mercury(II) Reductase	Stress	Metal	Mercury
	merP	Mercury Resistance System Periplasmic Binding Protein Merp	Stress	Metal	Mercury
	merT	Mercuric Transport Protein Mert	Stress	Metal	Mercury
	merR	Mercury Resistance Transcriptional Regulator Merr	Stress	Metal	Mercury
	qacL	Quaternary Ammonium Compound Efflux SMR Transporter Qacl	Stress	Biocide	Quaternary Ammonium
